# Impact of cognitive and linguistic ability on gaze behavior in children with hearing impairment

**DOI:** 10.3389/fpsyg.2013.00856

**Published:** 2013-11-18

**Authors:** Olof Sandgren, Richard Andersson, Joost van de Weijer, Kristina Hansson, Birgitta Sahlén

**Affiliations:** ^1^Department of Logopedics, Phoniatrics, and Audiology, Lund UniversityLund, Sweden; ^2^Humanities Laboratory, Lund UniversityLund, Sweden

**Keywords:** child hearing impairment, gaze behavior, referential communication, eye tracking, non-word repetition, phonological short term memory, Cox regression

## Abstract

In order to explore verbal–nonverbal integration, we investigated the influence of cognitive and linguistic ability on gaze behavior during spoken language conversation between children with mild-to-moderate hearing impairment (HI) and normal-hearing (NH) peers. Ten HI–NH and 10 NH-NH dyads performed a referential communication task requiring description of faces. During task performance, eye movements and speech were tracked. Cox proportional hazards regression was used to model associations between performance on cognitive and linguistic tasks and the probability of gaze to the conversational partner’s face. Analyses compare the listeners in each dyad (HI: *n* = 10, mean age = 12; 6 years, SD = 2; 0, mean better ear pure-tone average 33.0 dB HL, SD = 7.8; NH: *n* = 10, mean age = 13; 7 years, SD = 1; 11). Group differences in gaze behavior – with HI gazing more to the conversational partner than NH – remained significant despite adjustment for ability on receptive grammar, expressive vocabulary, and complex working memory. Adjustment for phonological short term memory, as measured by non-word repetition, removed group differences, revealing an interaction between group membership and non-word repetition ability. Stratified analysis showed a twofold increase of the probability of gaze-to-partner for HI with low phonological short term memory capacity, and a decreased probability for HI with high capacity, as compared to NH peers. The results revealed differences in gaze behavior attributable to performance on a phonological short term memory task. Participants with HI and low phonological short term memory capacity showed a doubled probability of gaze to the conversational partner, indicative of a visual bias. The results stress the need to look beyond the HI in diagnostics and intervention. Acknowledgment of the finding requires clinical assessment of children with HI to be supported by tasks tapping phonological processing.

## INTRODUCTION

Children with hearing impairment (HI) often receive intervention based on the degree of their impairment. For children with impairments of sensorineural etiology, that is, impairments caused by cochlear or retrocochlear abnormalities, audiological intervention ranges from cochlear implantation for the most severely impaired, to conventional type hearing aids for mild-to-moderate impairments. Educational intervention ranges from segregated schooling in classes for the deaf, with or without spoken language as the main mode of communication, to fully mainstreamed education in classes with normal-hearing (NH) peers. Increasingly, mainstreaming is promoted by educational authorities, and the preferred choice of many parents of children with HI. However, while differing greatly between schools, measures taken to support learning in mainstreamed children with HI can be limited to hearing assistive technology systems, such as microphones and radio-frequency transmission systems, while deeper knowledge of the consequences of a HI may be lacking. To manage the increasing demands on independence in school work and peer interactions, children with HI should be provided intervention targeting the broader range of cognitive and linguistic functions known to influence language development. Thus, there is a need to study the influence of the cognitive and linguistic aspects of HI on everyday functioning in a hearing environment.

### LANGUAGE AND COGNITION IN CHILDREN WITH HEARING IMPAIRMENT

The spoken language development of children with bilateral sensorineural hearing impairment (SNHI) has repeatedly been found to depart from the typical trajectory (Davis et al., 1986; Yoshinaga-Itano et al., 1998; Blamey et al., 2001; Wake et al., 2004), albeit with great individual variation. Many studies have failed to find a linear relationship between degree of HI and the level of language problems exhibited (Blamey et al., 2001; Hansson et al., 2004; Pittman et al., 2005). Davis et al. (1986) and Wake et al. (2004) found mild SNHI to be sufficient to cause a lag in vocabulary development, whereas Mayne et al. (1998a, b), using parent report, found significant delays in the early oral language development of both receptive and expressive vocabulary (signed communication was not included in the assessment) in children with impairments ranging from mild to severe. Using a novel word learning task, Gilbertson and Kamhi (1995) identified a subgroup with spared vocabulary skills among children with mild-to-moderate SNHI. The results suggested one subgroup developing typically despite their HI, and one with substantial language delay across a range of tasks, one being vocabulary acquisition. While confirming a difference between children with and without HI regarding novel word learning, Stelmachowicz et al. (2004) found no support of a subgroup without affected language ability. Current vocabulary size was found to be an important predictor of the ability to learn new words, with larger vocabularies facilitating additional vocabulary growth (Stelmachowicz et al., 2004).

With vocabulary emerging as a particularly vulnerable language domain in children with SNHI^1^, a number of studies have examined phonological processing as a possible origin of these deficits. Results are almost unanimous. Children with SNHI consistently perform below age norms on tasks requiring phonological sensitivity (for example, identification and manipulation of speech sounds) and phonological short term memory (often measured with non-word repetition, that is, repetition of phonologically plausible, yet non-existent, non-sense words; Briscoe et al., 2001; Sahlén et al., 2004; Wake et al., 2006). Wake et al. (2006) found deficits in phonological short term memory and phonological discrimination in a study of elementary school children with mild SNHI. Similar findings had previously been shown by Briscoe et al. (2001), later replicated by Sahlén et al. (2004), who found primary school children with mild-to-moderate SNHI to be as impaired as children with specific language impairment (SLI) on measures of phonological discrimination, phonological awareness, and phonological short term memory. Interestingly, despite substantial difficulties with phonological processing, the children with HI performed on par with typically developing NH peers on more global measures of language proficiency, with the exception of vocabulary. This suggests that phonological processing, which is generally seen as tightly linked to language development, appears to be more separate from other language abilities in children with mild-to-moderate SNHI (Briscoe et al., 2001; Wake et al., 2006). Similarly, studies of verbal working memory, that is, concurrent storing and processing of information, have failed to find differences in performance between children with and without HI (Hansson et al., 2004; Stiles et al., 2012b), thus linking the lag in vocabulary development displayed by children with SNHI to phonological deficits rather than differences in working memory capacity. Other studies have, however, found more pervasive effects of HI on additional language domains. Delage and Tuller (2007) found that over 60% of participating adolescents with mild-to-moderate SNHI performed below -1.65 SD on a phonological task (word repetition) and over 30% performed below the same level on a combined score of expressive and receptive grammar. The authors concluded that the language proficiency of children with mild-to-moderate SNHI does not normalize with age, and that the language domains still affected in adolescence are formal aspects of language functioning thought to be particularly vulnerable to impoverished input during the critical period of early language development (Delage and Tuller, 2007).

### AUDITORY-VISUAL INTEGRATION

Language is, however, multimodal and not perceived exclusively through the auditory modality. Speakers and listeners, with or without HI, continuously monitor facial signals of communication (including lip movements, facial gestures, gaze gestures, and gaze direction) to assist language processing. Often studied within the context of speechreading, predominantly in individuals with severe-to-profound HIs, this ability has recently been found to be equally developed in children and adolescents with and without HI (Kyle et al., 2013), although earlier studies have found an advantage for individuals with HI over peers with NH in child (Lyxell and Holmberg, 2000; studying children with moderate HIs) and adult (Mohammed et al., 2006) populations. Better speechreading has been seen as the consequence of a greater reliance on visual cues to access spoken language, similar for younger children of all degrees of hearing, but persisting among adolescents and adults with HI (Kyle et al., 2013). Furthermore, speechreading ability has been connected to phonological ability (Mohammed et al., 2006), as shown by a reduced speechreading ability in individuals with dyslexia, characterized by weak phonological representations (Mohammed et al., 2006; Kyle et al., 2013). However, phonological ability may contribute differently to speechreading ability for different groups and results indicate top–down processing, for example, using word or sentence level information to derive phonological details, to be used to a higher extent by individuals with HI than by NH speechreaders who, instead, use bottom–up processes to join together the visual representation of phonemes to infer word meaning (Mohammed et al., 2006). This more holistic approach in individuals with HI corresponds to findings of a greater use of visual/orthographic decoding strategies in children and adolescents with severe-to-profound HI and cochlear implants (Wass et al., 2010), suggested to bypass their reduced phonological ability and thereby limiting the negative impact on reading skills. The results paint a complex picture of a visual bias for children and adolescents with HI, likely to increase in cognitively demanding contexts – in reading as well as in conversation – more often encountered by older children and adults. In conversation, demanding contexts call on listeners, hearing impaired or not, to use all available sources of linguistic information to access the spoken message, as recently demonstrated by adverse listening conditions evoking similarly increased use of visual information to interpret a verbal message among participants with NH as in individuals with HI performing the same task in silence (Obermeier et al., 2012).

To summarize, the results of previous studies point to an important contribution of phonological processing to the language development of children with mild-to-moderate SNHI. The HI causes an auditory-perceptual deficit, resulting in imprecise phonological representations, which, in turn, hampers vocabulary acquisition and, to some extent, grammatical development. The absence of a direct link between the degree of HI and language proficiency suggests one or several factors modulating the effect of the HI. Blamey et al. (2001) suggest such factors to be a combination of, 1: environmental aspects, for example, quality and quantity of input, teaching, and feedback, 2: intrinsic factors such as cognitive abilities, for example, working memory capacity, and, 3: paralinguistic strategies acquired to aid language processing and comprehension, for example, the use of visual information. These factors can compensate for the degraded auditory input and the restricted ability to use incidental hearing for learning (Blamey et al., 2001).

### AIMS OF THE PRESENT STUDY

This study investigates the influence of cognitive and linguistic ability on gaze behavior as an indicator of a paralinguistic strategy active during conversation. Specifically, we study the effect of mild-to-moderate sensorineural HI on the probability of gaze to the conversational partner’s face, when adjusting for individual performance on measures of complex working memory, phonological short term memory, reception of grammar, and expressive vocabulary. In line with Blamey et al. (2001), we regard gaze to the conversational partner’s face as a possible compensatory strategy, likely to be used to a varying extent depending on individual and task characteristics. The present study uses an unscripted referential communication task to be performed with a NH peer. In contrast to many previous studies (Briscoe et al., 2001; Hansson et al., 2004; Stelmachowicz et al., 2004; Wake et al., 2004; Stiles et al., 2012b), data is reported on participants in middle childhood, a period of increasing demands on independence in school work and peer interactions.

With gaze and speech highly coordinated in conversation (Bavelas et al., 2002) we expect all participants, hearing impaired or not, to gaze to the conversational partner’s face, yet predict higher probability of gaze-to-partner in children with HI as an expression of an increased use of a visual compensatory strategy.

## MATERIALS AND METHODS

### PARTICIPANTS

#### HI–NH dyads

Twenty children, 7 and 13 boys, ranging in age between 9; 8 and 15; 10 years (mean 12; 4, SD = 1; 9) were recruited to form conversational pairs. Ten participants, 3 girls and 7 boys (mean age 12; 6 years, SD = 2; 0, labeled HI), had bilateral mild-to-moderate sensorineural HI, with better ear pure-tone average ranging between 20 and 43 dB HL (mean 33.0, SD = 7.8), and had received bilateral hearing aids. All impairments were symmetrical (mean difference between ears 7.1 dB, SD = 6.1). According to medical records, mean age at identification of the HI was 3; 7 years (SD = 1; 1) and mean age at amplification was 5; 2 years (SD = 2; 7). All participants with HI were raised in oral speaking families and were educated in oral settings, and given no formal training in sign language, visually aided communication, or speechreading. No participant showed phonological or articulatory errors in spontaneous speech production, corroborating earlier results (Hansson et al., 2007).

Remaining 10 participants, 4 girls and 6 boys (mean age 12; 3 years, SD = 1; 7) were NH same-age peers invited by the participant with HI to take part in the study as conversational partners. All participants with HI chose to bring a classmate, thus, a partner familiar with their hearing loss, differing maximally 1 year in age. All except three HI–NH dyads consisted of same-sex participants.

#### NH–NH dyads

Twenty children, 10 girls and 10 boys, ranging in age between 10; 2 and 15; 4 (mean 13; 6 years, SD = 1; 11) were recruited to form NH control dyads. Half of the participants in the control dyads, five girls and five boys (mean age 13; 7 years, SD = 1; 11, labeled NH) composed a control group, matched to the age of the HI group. The other half, five girls and five boys (mean age 13; 5 years, SD = 2; 0), were classmates invited by their NH peers to participate as conversational partners. All NH–NH dyads consisted of same-sex participants.

The HI and NH groups did not differ significantly on age [*t*(18) = 1.281, *p* = 0.22]. All participants had non-verbal IQ within normal limits (±1 SD) as measured by Raven’s Standard Progressive Matrices (Raven et al., 2004). All participants had normal or corrected to normal vision and all NH participants passed a 20 dB pure tone hearing screening at 0.5, 1, 2, 4, and 6 kHz before data collection. All participants had Swedish as their first language.

Written informed consent was gained from parents of all participants. Ethical approval for the study was granted by the Regional Ethics Review Board for southern Sweden, approval number 2009/383.

### PROCEDURE

#### Experimental task

An unscripted referential communication task was used, in which the conversational partners acted as the speaker, and the children with HI and the NH controls as the listener. This study reports data on the listener. The task has previously been developed and used in studies of conversational strategies and interaction in children with language and HI (Ibertsson et al., 2009; Sandgren et al., 2011) and in a study of gaze behavior in children with NH (Sandgren et al., 2012). A screen displaying 16 pictures of faces, visible only to the speaker, was placed between the participants. The listener was provided with 24 pictures of faces. The speaker was instructed to describe each picture and its position with enough detail for the listener to correctly identify the picture and its position. The pictures of faces differed only in details and the listener was forced to request further information when confronted with an insufficiently detailed description.

#### Equipment and data collection

While performing the task the participants wore identical SMI iView X HED head-mounted video based pupil and corneal reflection eye tracking systems (data on speaker gaze behavior to be reported elsewhere). The eye tracking systems were calibrated with a nine-point calibration procedure before data collection and continuously monitored for calibration deviations during the experiment. The data from each eye tracking system were superimposed the video of a forward-facing camera, creating an output video showing the participant’s field of view with a moving cursor indicating gaze position. The video was filmed at 25 frames/s, creating an effective sampling frequency of 25 Hz. The participants were seated at approximately 120 cm distance from each other, separated by the 30 cm-tall picture screen. The height of the screen allowed eye contact and visual cues. The dialogs were video-recorded using a fixed digital video camera capturing both participants from a side view. For audio-recording, the camera’s built-in microphone was used. Recordings were made in a quiet laboratory setting in the Humanities Laboratory at Lund University.

The dialogs were transcribed orthographically by the first author and transcriptions exported to ELAN (Wittenburg et al., 2006) where the listener’s speech was categorized according to the type of communicative event performed. Communicative events were categorized into four categories; requests, non-requests, back channeling, and listening. In all, 2946 cases of communicative events were identified and used in subsequent analyses. The fourth author independently coded the communicative events in 25% of the dialogs. The interrater reliability as estimated with Cohen’s Kappa was 0.941. **Table 1** provides examples and group data on each communicative event type.

**Table 1:** Communicative event types, descriptions, examples, and distribution.

Communicative event type	Description	Example	*n* (HI)	*n* (NH)
Requests	Questions	“Has she got blue eyes?”	288	254
		“What color are her eyes?”		
Non-requests	Statements	“He looks a bit like your dad”	176	309
Back-channeling	Feedback	“Uh-huh,” “Mhm”	269	165
Listening	Partner	–	745	740
	speaking			
Total *n*			1478	1468

Annotation of eye movements was made by the first author using ELAN (Wittenburg et al., 2006). The output videos of the eye tracking systems were merged and synchronized with the orthographic transcription, creating a combined annotation file of the listener’s gaze focus and communicative events. Three areas of interest regarding gaze focus were specified; Task (the pictures of faces), Face (the speaker’s face), and Off (gaze focused elsewhere). All instances of gaze within the specified areas of interest were recorded, providing information on the participants’ gaze focus for the duration of the conversation. On occasion, manual recoding was necessary due to slight calibration error. However, with large areas of interest gaze location could safely be determined, as exhibited by the high interrater reliability. The second author independently annotated the eye movements of both participants in 20% of the data and reliability was computed as percentage overlapping transcription annotations, using a weighted correction taking into account the duration of the annotation. The interrater reliability was 88.5%. The communicative event types have previously been shown to pattern qualitatively similarly with regard to gaze behavior, however, with minor quantitative differences expressing a higher probability of gaze-to-partner in association with the onset of requests compared to the onset of non-requests, and with the onset of back channeling compared to listening. Furthermore, an increased probability of gaze-to-partner has been found for children with SNHI, as compared to NH peers, across all communicative event types (Sandgren et al., 2013). Non-significant differences between the gaze distributions for speech events (requests, non-requests, and back channeling) and listening (Mantel–Cox log rank χ^2^ = 2.466, *p* = 0.116) warrant merging all communicative event types in the present analyses.

#### Cognitive and linguistic tests

In addition to the referential communication task, tests of cognitive and linguistic ability were administered to the target HI and NH groups. CLPT (Competing Language Processing Task; Gaulin and Campbell, 1994, Swedish adaptation) was used to assess complex working memory. TROG-2 (Test for Reception of Grammar – Second edition; Bishop, 2003, Swedish adaptation; Bishop, 2009) was used to assess receptive grammar, and BNT (Boston Naming Test; Kaplan et al., 2001, Swedish adaptation; Brusewitz and Tallberg, 2010) to test expressive vocabulary. Phonological short term memory was measured with a non-word repetition task, NWrep (Sahlén et al., 1999; Wass et al., 2008), assessing repetition ability of non-words of increasing length and complexity.

The testing lasted approximately 1 h and tests were administered in a fixed order: 1. CLPT; 2. NWrep; 3. TROG-2; 4. BNT. The results on TROG-2 were processed as percentiles, in accordance with standardized test procedure. For all other tests percent correct responses were computed.

#### Statistical analysis

Gaze and verbal annotation data were extracted from ELAN (Wittenburg et al., 2006) for analysis. The dependent variable (listener’s gaze to the speaker’s face) was scored binarily, on 10 ms intervals, over a 3000 ms time window centered at the onset of each communicative event. Thus, for each instance of a communicative event 300 measurements of the occurrence of gaze-to-partner (1/0) were made, covering the time span between 1500 ms preceding and 1500 ms following the communicative event onset. The choice to study gaze-to-partner as an effect of a triggering communicative event focuses the analysis on the part of the utterance most related to gaze exchanges between interlocutors. This method has advantages over, for example, averaging gaze to areas of interest over an entire utterance, which risks obscuring brief effects, thereby increasing the risk of type II errors. The size of the time window was chosen to accommodate natural variation between participants in utterance planning of conversation and timing of gaze-to-partner (Griffin and Bock, 2000), and to allow analysis of the probability of gaze-to-partner leading up to, and following, the communicative events. Data from overlapping time windows (that is, instances where two communicative events of the same type occurred within 3000 ms) were deemed not to affect the computations and were included in the analyses.

In order to answer not only if, but also when, gaze to the speaker’s face occurs data were fitted to a survival function estimating the probability of the target event to occur (gaze to the speaker’s face) when controlling for possibly influencing covariates. The survival function estimates the event time, that is, the time from the beginning of measurements to the target event, while statistically accommodating the influence of censored cases, that is, communicative events performed without gaze-to-partner within the time window, thereby yielding an estimate of changes in the probability of target event occurrence as a function of time. Cox proportional hazards regression was performed to model the hazard ratio (HR) of gaze to the speaker’s face after adjustment for the effect of group, and cognitive and linguistic covariates. Chi-square statistics were used to evaluate model change as covariates were added to a baseline model with group entered as the only covariate.

## RESULTS

Cox proportional hazards regression was performed to assess differences in gaze behavior between children with HI and NH peers after adjustment for the effect of cognitive and linguistic covariates known to influence language development in children with SNHI; receptive grammar (TROG-2), expressive vocabulary (BNT), complex working memory (CLPT), and phonological short term memory (NWrep). Age was not included as a covariate following preliminary analyses showing no relation with the dependent variable (gaze-to-partner) and non-significant differences between the groups. Independent samples *t*-tests revealed significant differences between the HI and NH groups on BNT [*t*(18) = 2.104, *p* = 0.05] and NWrep [*t*(18) = 3.274, *p* = 0.004], whereas non-significant group differences were found on TROG-2 [*t*(18) = 1.469, *p* = 0.159] and CLPT [*t*(18) = 1.417, *p* = 0.174]. **Table 2** presents descriptive data on included covariates.

**Table 2:** Descriptive statistics and test of group differences of covariates included in the Cox regression models.

Test	Group	Mean (SD)	Range	*p*
TROG-2^a^	HI	45.6 (17.6)	8–66	0.16
	NH	56.9 (16.8)	30–82	
BNT^b^	HI	76.7 (9.6)	60–86.7	0.05
	NH	84.3 (6.3)	75–91.7	
CLPT^c^	HI	62.6 (11.9)	50–85.7	0.17
	NH	71.2 (15.0)	38.1–90.5	
NWrep^d^	HI	51.3 (20.6)	20.8–79.2	0.004
	NH	76.7 (13.4)	58.3–95.8	

a Test for Reception of Grammar – Second edition.

b Boston Naming Test.

c Competing Language Processing Task.

d Non-word Repetition. Mean score and standard deviation in percentage correct except TROG-2 in percentiles.

*p value for test of difference between group with hearing impairment (HI) and normal-hearing (NH) peers.*

All instances of communicative events (*n* = 2946) were used as cases in the Cox regression models. Of the cases, 1825 (61.9%) were censored, that is, communicative events produced without gaze-to-partner within the specified 3000 ms time window. The proportion of censored cases was higher in the NH (68.3%) than in the HI group (55.6%).

The first Cox regression model was used as a baseline and entered Group as the only covariate. Group significantly predicted the probability of gaze-to-partner [χ^2^(1) = 47.29, *p* < 0.0005] with HI showing a 51% probability increase, compared to NH (HR = 1.51, 95% CI: 1.34–1.70, *p* < 0.0005). As a first step of analysis the effect of Group was investigated while adjusting for the other covariates separately. Adjustment for TROG-2, BNT, and CLPT only marginally affected the effect of Group. Adjustment for TROG-2 increased the HR somewhat [χ^2^(2) = 67.45, *p* < 0.0005; HR = 1.68, 95% CI: 1.47–1.90, *p* < 0.0005], whereas BNT [χ^2^(2) = 55.61, *p* < 0.0005; HR = 1.33, 95% CI: 1.15–1.54, *p* < 0.0005] and CLPT [χ^2^(2) = 48.76, *p* < 0.0005; HR = 1.46, 95% CI: 1.28–1.66, *p* < 0.0005] decreased it slightly. The effect of Group was also left largely unaffected when adjusting for TROG-2, BNT, and CLPT in a single step [χ^2^(4) = 80.15, *p* < 0.0005; HR = 1.45, 95% CI: 1.24–1.70, *p* < 0.0005].

Group adjusted for NWrep significantly predicted the probability of gaze-to-partner [χ^2^(2) = 107.88, *p* < 0.0005], although removing the significance of Group [HR = 1.04, 95% CI: 0.89–1.21, *p* = 0.65]. Instead, NWrep significantly contributed to the model (HR = 0.986, 95% CI: 0.982–0.989, *p* < 0.0005).

The loss of the effect of Group when adjusting for NWrep, as well as the significant contribution to the model of NWrep, called for a closer examination of a possible interaction between non-word repetition ability and HI. The second model investigated the effect of Group while adjusting for NWrep and the Group × NWrep interaction. The model was significant [χ^2^(3) = 122.22, *p* < 0.0005] and the adjustment substantially increased the effect of Group on the probability of gaze-to-partner (HR = 3.16, 95% CI: 1.73–5.78, *p* < 0.0005). The interaction term contributed significantly to the model (HR = 0.984, 95% CI: 0.975–0.992, *p* < 0.0005).

The third model investigated the effect of Group while adjusting for all covariates, including the Group × NWrep interaction. The model as a whole was significant [χ^2^(6) = 162.01, *p* < 0.0005) and showed an almost threefold increase in the probability of gaze-to-partner for participants with HI (HR = 2.86, 95% CI: 1.50–5.47, *p* = 0.001), when adjusting for all other covariates.

Since the interaction between Group and non-word repetition ability contributed significantly to the model and, in addition, had a large impact on the HR of Group, a final analysis investigated the effect of Group on the probability of gaze-to-partner as a function of the level of NWrep performance. The participants were divided into high and low performance on their NWrep scores. The cut-off was set to 1.25 SD below the mean of the NH group, following diagnostic recommendations (Tomblin et al., 1996), corresponding to 60% non-words correctly repeated. Seven participants, six children with HI and one child with NH, performed below the cut-off score. These participants contributed 1053 communicative events (51.9% censored) to the analysis. The high performers consisted of 13 participants, 4 children with HI and 9 NH participants, contributing 1893 communicative events (67.5% censored).

A Cox regression – stratified on NWrep performance – with Group, TROG-2, BNT, and CLPT as covariates, significantly predicted the probability of gaze-to-partner for both low [χ^2^(4) = 56.67, *p* < 0.0005] and high [χ^2^(4) = 51.06, *p* < 0.0005] NWrep performers. Participants with HI scoring low on the NWrep task exhibited a more than twofold increase in the probability of gaze-to-partner (HR = 2.17, 95% CI: 1.58–2.98, *p* < 0.0005), whereas children with HI and high non-word repetition performance had a decreased probability of gaze-to-partner (HR = 0.67, 95% CI: 0.50–0.90, *p* = 0.008). With the exception of CLPT in the high NWrep performance group, TROG-2, BNT, and CLPT made significant (at *p* = 0.01), albeit minor, contributions to the model.

**Table 3** presents hazard ratios (with confidence intervals), and p values for the effect of Group on the probability of gaze-to-partner, for the different steps of adjustment.

**Table 3:** Hazard ratios and *p* values for the effect of Group on the probability of gaze-to-partner, for the different steps of adjustment.

Contrast	*n*	HR (95% CI)	*p*	*p* interaction
Group^1^	2946	1.51 (1.34–1.70)	<0.0005	
Group^2^		1.45 (1.24–1.70)	<0.0005	
Group^3^		3.16 (1.73–5.78)	<0.0005	<0.0005
Group^4^		2.86 (1.49–5.47)	0.001	<0.0005
Low NWrep^4b^	1053	2.17 (1.58–2.98)	<0.005	
High NWrep^4b^	1893	0.67 (0.50–0.90)	0.008	

1 Model adjusted for Group.

2 Model adjusted for Group, TROG-2, BNT, CLPT.

3 Model adjusted for Group, NWrep, Group × NWrep.

4 Model adjusted for Group, TROG-2, BNT, CLPT, NWrep, Group × NWrep.

4b p Model adjusted for Group, TROG-2, BNT, CLPT stratified on NWrep performance. HR presents hazard ratio estimates for HI (with 95% confidence intervals).

*values present significance of contribution to the model for Group, and Group × NWrep interaction.*

To summarize the results, group differences in gaze behavior were found, with HI showing higher probability of gaze-to-partner than NH. The effect of Group withstood adjustment for receptive grammar, expressive vocabulary, and complex working memory, but not non-word repetition, revealing an interaction between HI and phonological short term memory. Participants with HI and low phonological short term memory capacity showed a twofold increase in the probability of gaze-to-partner.

## DISCUSSION

This study provides evidence for an explanatory role of cognitive and linguistic functioning on gaze behavior during conversation in children with mild-to-moderate SNHI. We report group differences regarding the use of gaze to the conversational partner’s face which go above and beyond what is explained by the HI alone, and highlight phonological short term memory capacity as the principal driving force behind the effect. The results suggest areas of improvement in clinical identification and assessment, as well as educational intervention, of children with SNHI.

The present sample of children with SNHI performed significantly below NH peers on non-word repetition and expressive vocabulary, while non-significant differences were found regarding receptive grammar and complex working memory. This agrees with previous research pointing out phonology and vocabulary as main areas of deficit in children with SNHI (Davis et al., 1986; Gilbertson and Kamhi, 1995; Briscoe et al., 2001; Wake et al., 2006; Delage and Tuller, 2007; Stiles et al., 2012a), while reporting receptive grammar and working memory less likely to be affected (Gilbertson and Kamhi, 1995; Briscoe et al., 2001; Stiles et al., 2012b). As suggested by Sahlén and Hansson (2006), this implies a continued strong link between phonology and vocabulary in children with SNHI in middle childhood. This contrasts to NH children of the same age for whom phonological processing no longer reliably predicts more complex language abilities (Hesketh and Conti-Ramsden, 2013).

The proportion of censored cases in each group, that is, communicative events performed without gaze-to-partner, confirmed the first part of our hypothesis; gaze to the conversational partner’s face occurs frequently among all participants, hearing impaired or not. This finding corroborates previous studies on the integration of speech and gaze in interaction. Using a storytelling task, Bavelas et al. (2002) studied the microstructure of interaction and found gaze-to-partner to enable speaker change, as well as short intervals of feedback from the partner. Interpreted within the context of this study, gaze to the conversational partner in conjunction with a request, for example, could, similarly, signal the ensuing speaker change.

The second part of our hypothesis, that the participants with SNHI would display a higher probability of gaze-to-partner, was also confirmed. The initial analysis showed a 51% increase of the probability of gaze to the conversational partner’s face for HI compared to NH. But what drives this increased probability? Our analyses provide evidence that individual ability on measures of expressive vocabulary, receptive grammar, and complex working memory has little to do with the increase. Non-word repetition, on the other hand, has a great influence on the probability, removing the significant effect of group. However, when taking into account the interaction between HI and non-word repetition ability the effect of group is substantially increased. Finally, when dividing the participants on their non-word repetition performance, those with HI and low non-word repetition scores (less than 60% non-words correctly repeated) displayed a doubled probability of gaze-to-partner, while those with SNHI and high scores (more than 60% correct) had a reduced probability, compared to NH peers.

But what is so special about non-word repetition? And why does it influence the probability of gaze to the partner’s face during conversation? In children with NH and typical language development non-word repetition has been shown to eventually lose its power to predict more general language abilities (Gathercole, 1999; Hesketh and Conti-Ramsden, 2013). This transition has not been shown for children with SLI for whom non-word repetition continues to be an important predictor in middle childhood (Hesketh and Conti-Ramsden, 2013). The reason suggested is that, during the course of language development, the relative contribution of the underlying abilities necessary for non-word repetition [short term memory, phonological representation, encoding and retrieval, and phonological output (Bowey, 2006)] changes, from phonological representation to short term memory being the stronger predictor of non-word repetition ability (Rispens and Baker, 2012). Consequently, non-word repetition appears to be an increasingly cognitive task. However, the predictive power of non-word repetition on general language abilities in children with SLI indicates that the task, for this group, continues to tax language functioning, thus making non-word repetition ability vulnerable to several cognitive and linguistic deficits (Graf Estes et al., 2007). Although the underlying causes may differ, the surface similarities between children with SNHI and SLI regarding non-word repetition suggest similar developmental trajectories. As such, even in middle childhood the children with SNHI may find the referential communication task used in the present study taxing enough to call for use of additional available sources of information, for example, gaze to the conversational partner.

### IMPLICATIONS AND FUTURE STUDIES

With its long-lasting impact on the language functioning of children with SNHI, phonological short term memory should be routinely assessed, and, if found to be affected, targeted through intervention of phonology and vocabulary. Although etiologically linked to the HI, cognitive and linguistic aspects still influencing communicative behavior in middle childhood should be the target of direct intervention, especially when appropriate audiological intervention and hearing assistive technology systems have been provided. Intervention should focus on phonological processing within cognitively demanding contexts, for example, conversation. With training on the use of verbal and non-verbal means in conversation, for example, through use of referential communication tasks, children with HI and limited phonological short term memory capacity are likely to benefit more from the linguistic input of face-to-face conversation. However, the intervention must be based on individual needs. As previously pointed out by Gilbertson and Kamhi (1995), there are risks associated both with assuming language problems in all children with SNHI, and with assuming no problems beyond the HI. Assuming problems in all would, admittedly, grant all those affected necessary intervention, but would also risk leading to lowered expectations and achievements for children with SNHI without language problems. Our results corroborate those of Gilbertson and Kamhi (1995) in revealing a subgroup among the participants with SNHI, performing within the normal range on non-word repetition and using visual cues to a lesser extent than NH peers. Future studies should investigate the effectiveness of non-word repetition as a possible screening method to identify those children with SNHI more likely to suffer adverse effects on language development, and thus, in need of language intervention.

This study has highlighted the need to look beyond the HI in order to correctly evaluate its effect. Increased probability of gaze to the conversational partner’s face should not be regarded as simply a problem of signal transfer but as a sign of the multimodal nature of conversation. However, without a measure of conversational success, we cannot with certainty claim that an increased probability of gaze-to-partner reflects an increased need for visual support. Although suggested by the higher probability among those with lower non-word repetition scores, the increased probability of gaze-to-partner could, alternatively, represent a habit, stemming from a previous need, or be the result of explicit training. A comparative study of gaze behavior in SNHI and NH children with SLI, with comparable deficits in phonological short term memory, could help determine whether gaze-to-partner compensates for degraded auditory input or limitations in phonological processing, especially with the addition of a measure of pragmatic functioning, an area known to be affected in SLI, but not in SNHI. Future studies should also investigate the influence of the partner on gaze behavior. Results indicating reduced levels of gaze to opposite sex partners (Turkstra, 2001) call for a replication of the present study supplemented with systematic variation of the conversational partners.

## Conflict of Interest Statement

The authors declare that the research was conducted in the absence of any commercial or financial relationships that could be construed as a potential conflict of interest.

## Footnotes

1 However, please note that the vocabulary development of deaf children acquiring sign language follows a normal trajectory (see, for example, Morgan and Woll, 2002).
